# 3-Cyano-*N*-(2-hydroxy­benz­yl)anilinium chloride

**DOI:** 10.1107/S1600536810003521

**Published:** 2010-02-03

**Authors:** Jing Dai, Wen-Ni Zheng

**Affiliations:** a Ordered Matter Science Research Center, College of Chemistry and Chemical Engineering, Southeast University, Nanjing 210096, People’s Republic of China

## Abstract

In the cation of the title compound, C_14_H_13_N_2_O^+^·Cl^−^, the two benzene rings are roughly parallel and are twisted slightly from each other by a dihedral angle of only 2.87 (1)°. In the crystal, weak inter­molecular N—H⋯Cl and O—H⋯Cl hydrogen bonds link the cations and anions into chains extended along the *b* axis.

## Related literature

For the crystal structures and properties of related compounds, see: Fu *et al.* (2007 ▶, 2008 ▶, 2009 ▶); Fu & Xiong (2008 ▶); Zhao *et al.* (2008 ▶); Loeb *et al.* (2005 ▶).
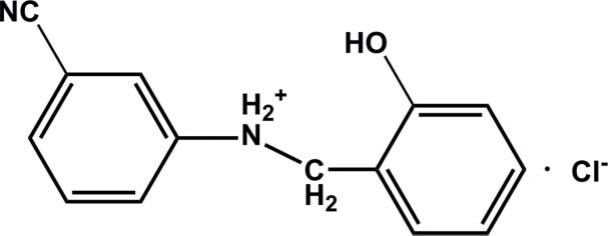


## Experimental

### 

#### Crystal data

C_14_H_13_N_2_O^+^·Cl^−^
*M* *_r_* = 260.71
Monoclinic, 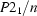

*a* = 13.071 (3) Å
*b* = 7.9437 (16) Å
*c* = 13.141 (3) Å
β = 90.18 (3)°
*V* = 1364.4 (5) Å^3^
*Z* = 4
Mo *K*α radiation
μ = 0.27 mm^−1^
*T* = 298 K
0.4 × 0.35 × 0.2 mm

#### Data collection

Rigaku Mercury2 diffractometer
Absorption correction: multi-scan (*CrystalClear*; Rigaku, 2005 ▶) *T*_min_ = 0.881, *T*_max_ = 0.940
13632 measured reflections
3116 independent reflections
2264 reflections with *I* > 2σ(*I*)
*R*_int_ = 0.048

#### Refinement

*R*[*F* ^2^ > 2σ(*F* ^2^)] = 0.050
*wR*(*F* ^2^) = 0.127
*S* = 1.05
3116 reflections
163 parameters
3 restraints
H-atom parameters constrained
Δρ_max_ = 0.31 e Å^−3^
Δρ_min_ = −0.26 e Å^−3^

### 

Data collection: *CrystalClear* (Rigaku, 2005 ▶); cell refinement: *CrystalClear*; data reduction: *CrystalClear*; program(s) used to solve structure: *SHELXS97* (Sheldrick, 2008 ▶); program(s) used to refine structure: *SHELXL97* (Sheldrick, 2008 ▶); molecular graphics: *SHELXTL* (Sheldrick, 2008 ▶); software used to prepare material for publication: *SHELXTL*.

## Supplementary Material

Crystal structure: contains datablocks I, global. DOI: 10.1107/S1600536810003521/dn2531sup1.cif

Structure factors: contains datablocks I. DOI: 10.1107/S1600536810003521/dn2531Isup2.hkl

Additional supplementary materials:  crystallographic information; 3D view; checkCIF report

## Figures and Tables

**Table 1:** Hydrogen-bond geometry (Å, °)

*D*—H⋯*A*	*D*—H	H⋯*A*	*D*⋯*A*	*D*—H⋯*A*
N1—H1*A*⋯Cl1^i^	0.90	2.29	3.1315 (17)	155
O1—H1⋯Cl1^ii^	0.85	2.24	3.0870 (18)	171
N1—H1*B*⋯Cl1	0.90	2.14	3.0376 (16)	173

Symmetry codes: (i) 
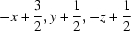
; (ii) 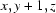.

**Fig. 1.**
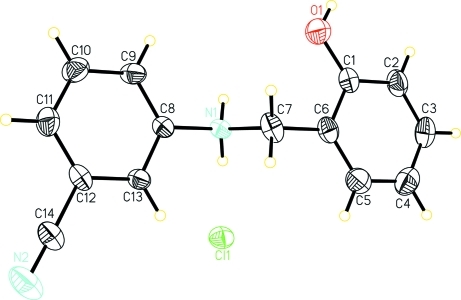
A view of the title compound with the atomic numbering scheme. Displacement ellipsoids were drawn at the 30% probability level.

**Fig. 2.**
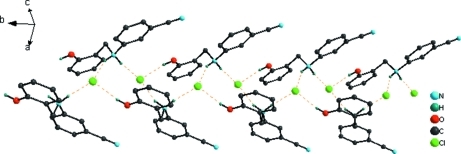
The crystal packing of the title compound, showing the one-dimensional hydrogen-bonded chain. H atoms not involved in hydrogen bonding (dashed line) have been omitted for clarity.

## supplementary crystallographic information

### Comment

In the last few years, more and more people have focused on the chemistry of nitrile derivatives because of their wide range of applications in industry and coordination chemistry as ligands (Fu *et al.*, 2007; Fu & Xiong 2008). For example, phthalonitriles have been used as starting materials for phthalocyanines, which are important components for dyes, pigments, gas sensors, optical limiters and liquid crystals, and which are also used in medicine, as singlet oxygen photosensitisers for photodynamic therapy. Recently, we have reported a few benzonitrile compounds (Zhao *et al.*, 2008; Fu *et al.*, 2008; Fu *et al.*, 2009). As an extension of our work on the structural characterization, we report here the crystal structure of the title compound, 3-cyano-*N*-(2-hydroxybenzyl)anilinium chloride.

In the title compound (Fig.1), the amino N atom is protonated. The phenyl rings are roughly parallel and only slightly twisted from each other by a dihedral angle of 2.87 (1) °. A larger twist angle of 20.7 (3)° is observed in the related N-(4-(Trifluoromethyl)benzyl)-4-methoxyanilinium trifluoromethanesulfonate compound (Loeb *et al.*, 2005). The nitrile group bond length of 1.127 (2)Å is within the normal range.

The crystal packing is stabilized by N—H···Cl and O—H···Cl hydrogen bonds to form a one-dimensional chain parallel to the *b* axis. (Table 1, Fig. 2).

### Experimental

The commercial 3-(2-hydroxybenzylamino)benzonitrile (3 mmol, 669 mg) was dissolved in water/HCl (50:1 *v*/*v*) solution. The solvent was slowly evaporated in air affording colourless block-shaped crystals of the title compound suitable for X-ray analysis.

While the permittivity measurement shows that there is no phase transition within the temperature range (from 100 K to 400 K), and the permittivity is 9 at 1 MHz at room temperature.

### Refinement

All H atoms attached to C atoms were fixed geometrically and treated as riding with C–H = 0.93 Å (aromatic) and C–H = 0.97 Å (methylene) with *U*_iso_(H) = 1.2*U*_eq_(C or N). H atoms attached to O and N atoms located in difference Fourier maps and freely refined. In the last stage of refinement they were treated as riding on the O and N, with *U*_iso_(H) = 1.5*U*_eq_(O and N).

### Crystal data

**Table:**

C_14_H_13_N_2_O^+^·Cl^−^	*F*(000) = 544
*M**_r_* = 260.71	*D*_x_ = 1.269 Mg m^−^^3^
Monoclinic, *P*2_1_/*n*	Mo *K*α radiation, λ = 0.71073 Å
Hall symbol: -P 2yn	Cell parameters from 2264 reflections
*a* = 13.071 (3) Å	θ = 3.0–27.5°
*b* = 7.9437 (16) Å	µ = 0.27 mm^−^^1^
*c* = 13.141 (3) Å	*T* = 298 K
β = 90.18 (3)°	Block, colourless
*V* = 1364.4 (5) Å^3^	0.4 × 0.35 × 0.2 mm
*Z* = 4	

### Data collection

**Table:**

Rigaku Mercury2 diffractometer	3116 independent reflections
Radiation source: fine-focus sealed tube	2264 reflections with *I* > 2σ(*I*)
graphite	*R*_int_ = 0.048
Detector resolution: 13.6612 pixels mm^-1^	θ_max_ = 27.5°, θ_min_ = 3.0°
ω scans	*h* = −16→16
Absorption correction: multi-scan (*CrystalClear*; Rigaku, 2005)	*k* = −10→10
*T*_min_ = 0.881, *T*_max_ = 0.940	*l* = −17→17
13632 measured reflections	

### Refinement

**Table:**

Refinement on *F*^2^	Primary atom site location: structure-invariant direct methods
Least-squares matrix: full	Secondary atom site location: difference Fourier map
*R*[*F*^2^ > 2σ(*F*^2^)] = 0.050	Hydrogen site location: inferred from neighbouring sites
*wR*(*F*^2^) = 0.127	H-atom parameters constrained
*S* = 1.05	*w* = 1/[σ^2^(*F*_o_^2^) + (0.0544*P*)^2^ + 0.2789*P*] where *P* = (*F*_o_^2^ + 2*F*_c_^2^)/3
3116 reflections	(Δ/σ)_max_ < 0.001
163 parameters	Δρ_max_ = 0.31 e Å^−^^3^
3 restraints	Δρ_min_ = −0.26 e Å^−^^3^

### Special details

**Table:**

Geometry. All e.s.d.'s (except the e.s.d. in the dihedral angle between two l.s. planes) are estimated using the full covariance matrix. The cell e.s.d.'s are taken into account individually in the estimation of e.s.d.'s in distances, angles and torsion angles; correlations between e.s.d.'s in cell parameters are only used when they are defined by crystal symmetry. An approximate (isotropic) treatment of cell e.s.d.'s is used for estimating e.s.d.'s involving l.s. planes.
Refinement. Refinement of *F*^2^ against ALL reflections. The weighted *R*-factor *wR* and goodness of fit *S* are based on *F*^2^, conventional *R*-factors *R* are based on *F*, with *F* set to zero for negative *F*^2^. The threshold expression of *F*^2^ > σ(*F*^2^) is used only for calculating *R*-factors(gt) *etc*. and is not relevant to the choice of reflections for refinement. *R*-factors based on *F*^2^ are statistically about twice as large as those based on *F*, and *R*- factors based on ALL data will be even larger.

### Fractional atomic coordinates and isotropic or equivalent isotropic displacement parameters (Å^2^)

**Table:**

	*x*	*y*	*z*	*U*_iso_*/*U*_eq_	
N1	0.64724 (11)	0.46075 (18)	0.35772 (11)	0.0389 (4)	
H1A	0.6623	0.5479	0.3166	0.058*	
H1B	0.6961	0.3821	0.3496	0.058*	
O1	0.70213 (12)	0.84372 (19)	0.41720 (13)	0.0677 (5)	
H1	0.7241	0.9398	0.3990	0.102*	
C12	0.42880 (16)	0.1649 (2)	0.33106 (15)	0.0474 (5)	
C13	0.52443 (14)	0.2279 (2)	0.35670 (14)	0.0420 (4)	
H13	0.5712	0.1625	0.3927	0.050*	
C8	0.54865 (14)	0.3902 (2)	0.32752 (14)	0.0389 (4)	
C11	0.35950 (16)	0.2619 (3)	0.27743 (17)	0.0559 (5)	
H11	0.2953	0.2190	0.2610	0.067*	
C9	0.48044 (15)	0.4880 (2)	0.27398 (17)	0.0499 (5)	
H9	0.4978	0.5972	0.2552	0.060*	
C6	0.74965 (16)	0.5918 (3)	0.49665 (15)	0.0487 (5)	
C1	0.77360 (16)	0.7568 (3)	0.47075 (16)	0.0494 (5)	
C7	0.64809 (17)	0.5202 (3)	0.46654 (16)	0.0552 (6)	
H7A	0.5957	0.6054	0.4753	0.066*	
H7B	0.6318	0.4265	0.5109	0.066*	
C2	0.86690 (18)	0.8245 (3)	0.50023 (18)	0.0611 (6)	
H2	0.8834	0.9346	0.4828	0.073*	
C10	0.38606 (16)	0.4227 (3)	0.24836 (19)	0.0603 (6)	
H10	0.3400	0.4877	0.2112	0.072*	
C14	0.40186 (18)	−0.0046 (3)	0.36042 (19)	0.0601 (6)	
C3	0.93473 (19)	0.7289 (4)	0.55504 (19)	0.0690 (7)	
H3	0.9973	0.7747	0.5745	0.083*	
C5	0.81903 (19)	0.4986 (3)	0.55185 (17)	0.0613 (6)	
H5	0.8032	0.3882	0.5693	0.074*	
C4	0.9113 (2)	0.5654 (4)	0.58170 (18)	0.0702 (7)	
H4	0.9573	0.5013	0.6194	0.084*	
N2	0.3796 (2)	−0.1368 (3)	0.3823 (2)	0.0900 (8)	
Cl1	0.79799 (4)	0.17211 (7)	0.33575 (4)	0.05632 (19)	

### Atomic displacement parameters (Å^2^)

**Table:**

	*U*^11^	*U*^22^	*U*^33^	*U*^12^	*U*^13^	*U*^23^
N1	0.0389 (8)	0.0327 (8)	0.0452 (9)	−0.0017 (6)	0.0049 (6)	−0.0003 (7)
O1	0.0616 (10)	0.0608 (10)	0.0808 (12)	−0.0066 (8)	−0.0118 (8)	0.0039 (8)
C12	0.0514 (12)	0.0390 (10)	0.0519 (12)	−0.0115 (9)	0.0063 (9)	−0.0078 (9)
C13	0.0455 (11)	0.0337 (9)	0.0469 (11)	−0.0002 (8)	0.0006 (8)	0.0020 (8)
C8	0.0361 (10)	0.0360 (9)	0.0447 (10)	−0.0011 (8)	0.0059 (8)	−0.0030 (8)
C11	0.0419 (11)	0.0632 (14)	0.0625 (13)	−0.0077 (10)	−0.0020 (10)	−0.0044 (11)
C9	0.0440 (12)	0.0403 (11)	0.0654 (13)	0.0024 (9)	0.0048 (9)	0.0091 (9)
C6	0.0529 (12)	0.0520 (12)	0.0412 (10)	−0.0106 (10)	0.0068 (9)	−0.0092 (9)
C1	0.0490 (12)	0.0506 (12)	0.0487 (11)	−0.0075 (10)	−0.0008 (9)	−0.0096 (10)
C7	0.0590 (13)	0.0570 (13)	0.0497 (12)	−0.0183 (10)	0.0132 (10)	−0.0139 (10)
C2	0.0628 (15)	0.0572 (13)	0.0634 (14)	−0.0205 (11)	−0.0077 (11)	−0.0055 (11)
C10	0.0432 (12)	0.0614 (14)	0.0762 (16)	0.0049 (10)	−0.0043 (10)	0.0102 (12)
C14	0.0666 (15)	0.0466 (13)	0.0671 (14)	−0.0145 (11)	0.0020 (11)	−0.0045 (11)
C3	0.0585 (15)	0.0872 (18)	0.0613 (14)	−0.0176 (13)	−0.0128 (11)	−0.0104 (13)
C5	0.0781 (17)	0.0551 (13)	0.0507 (12)	−0.0046 (12)	0.0055 (11)	0.0002 (10)
C4	0.0719 (17)	0.0850 (18)	0.0537 (14)	0.0066 (14)	−0.0082 (12)	0.0000 (13)
N2	0.111 (2)	0.0551 (13)	0.1037 (19)	−0.0313 (13)	0.0078 (15)	0.0019 (12)
Cl1	0.0555 (3)	0.0508 (3)	0.0628 (3)	0.0132 (2)	0.0059 (2)	−0.0056 (2)

### Geometric parameters (Å, °)

**Table:**

N1—C8	1.459 (2)	C6—C5	1.376 (3)
N1—C7	1.506 (3)	C6—C1	1.390 (3)
N1—H1A	0.9000	C6—C7	1.496 (3)
N1—H1B	0.9000	C1—C2	1.387 (3)
O1—C1	1.356 (3)	C7—H7A	0.9700
O1—H1	0.8500	C7—H7B	0.9700
C12—C11	1.381 (3)	C2—C3	1.370 (3)
C12—C13	1.387 (3)	C2—H2	0.9300
C12—C14	1.445 (3)	C10—H10	0.9300
C13—C8	1.382 (3)	C14—N2	1.127 (3)
C13—H13	0.9300	C3—C4	1.380 (4)
C8—C9	1.375 (3)	C3—H3	0.9300
C11—C10	1.378 (3)	C5—C4	1.373 (3)
C11—H11	0.9300	C5—H5	0.9300
C9—C10	1.379 (3)	C4—H4	0.9300
C9—H9	0.9300		
			
C8—N1—C7	112.49 (14)	O1—C1—C2	123.5 (2)
C8—N1—H1A	109.1	O1—C1—C6	116.84 (18)
C7—N1—H1A	109.1	C2—C1—C6	119.7 (2)
C8—N1—H1B	109.1	C6—C7—N1	111.97 (16)
C7—N1—H1B	109.1	C6—C7—H7A	109.2
H1A—N1—H1B	107.8	N1—C7—H7A	109.2
C1—O1—H1	111.7	C6—C7—H7B	109.2
C11—C12—C13	120.78 (18)	N1—C7—H7B	109.2
C11—C12—C14	119.76 (19)	H7A—C7—H7B	107.9
C13—C12—C14	119.5 (2)	C3—C2—C1	119.9 (2)
C8—C13—C12	118.45 (18)	C3—C2—H2	120.0
C8—C13—H13	120.8	C1—C2—H2	120.0
C12—C13—H13	120.8	C11—C10—C9	120.5 (2)
C9—C8—C13	121.38 (18)	C11—C10—H10	119.8
C9—C8—N1	119.52 (16)	C9—C10—H10	119.8
C13—C8—N1	119.06 (17)	N2—C14—C12	178.9 (3)
C10—C11—C12	119.56 (19)	C2—C3—C4	120.7 (2)
C10—C11—H11	120.2	C2—C3—H3	119.6
C12—C11—H11	120.2	C4—C3—H3	119.6
C8—C9—C10	119.37 (19)	C4—C5—C6	121.3 (2)
C8—C9—H9	120.3	C4—C5—H5	119.4
C10—C9—H9	120.3	C6—C5—H5	119.4
C5—C6—C1	119.2 (2)	C5—C4—C3	119.1 (2)
C5—C6—C7	121.2 (2)	C5—C4—H4	120.4
C1—C6—C7	119.6 (2)	C3—C4—H4	120.4

### Hydrogen-bond geometry (Å, °)

**Table:**

*D*—H···*A*	*D*—H	H···*A*	*D*···*A*	*D*—H···*A*
N1—H1A···Cl1^i^	0.90	2.29	3.1315 (17)	155
O1—H1···Cl1^ii^	0.85	2.24	3.0870 (18)	171
N1—H1B···Cl1	0.90	2.14	3.0376 (16)	173

Symmetry codes: (i) −*x*+3/2, *y*+1/2, −*z*+1/2; (ii) *x*, *y*+1, *z*.
